# Case report: Personalized management of treatment resistance in advanced NSCLC patients with mutated epidermal growth factor receptor: special examples and literature review

**DOI:** 10.3389/fonc.2025.1525881

**Published:** 2025-02-10

**Authors:** Jun Wang, Xiaojing Li, Shuang Dong, Sheng Hu, Fengming Ran, Yu Qian

**Affiliations:** ^1^ Department of Thoracic Oncology, Hubei Cancer Hospital, Tongji Medical College, Huazhong University of Science and Technology, Wuhan, China; ^2^ Laboratory of Natural Medicine and Molecular Engineering, College of Plant Science and Technology, Huazhong Agricultural University, Wuhan, China; ^3^ Department of Pharmacy, Puren Hospital, Wuhan University of Science and Technology, Wuhan, China

**Keywords:** anlotinib, adenocarcinoma, SCLC transformation, TKIs resistance, personalized management

## Abstract

The treatment landscape of non-small cell lung cancer (NSCLC) has shifted significantly from empirical, histology-driven, and clinician-directed cytotoxic regimens to a stratified approach predicated on molecular profiling of tumor genetics and immune biomarkers, by the former can indicate targeted therapy that bull’s eye hits the arrow, while the latter can hint the benefit amplitude of immune checkpoint inhibitors (ICBs). While third-generation epidermal growth factor receptor tyrosine kinase inhibitors (EGFR TKIs) have become the cornerstone of frontline therapy for patients harboring classic sensitive EGFR mutations, all tumors ultimately develop acquired resistance to these approaches, which can be categorized into three primary mechanism subclasses. The first subclass involves the acquisition of target mutations that lead to changes in the kinase domain, thereby hindering drug binding. The second mechanism, known as bypass resistance, entails tumor clones utilizing alternative signaling pathways for proliferation. Lastly, the third acquired mechanism pertains to histological transformation, such as the emergence of small cell lung cancer (SCLC) clones. The transformation of pathological types has brought great confusion to the clinical diagnosis and treatment process. We report a case of advanced lung adenocarcinoma with EGFR-sensitive mutation that transformed into small cell lung cancer after EGFR-TKIs treatment. Subsequent treatment revealed the presence of both adenocarcinoma and small cell carcinoma through needle biopsies at various metastatic sites. Based on the pathological, the patient received combination therapy with anlotinib at different times and achieved a long survival time.

## Introduction

The incidence and mortality of lung cancer are among the highest in China and the world (1). Non-small cell lung cancer (NSCLC) and small cell lung cancer (SCLC) are two common histopathologic types (2). Advanced NSCLC patients with epidermal growth factor receptor (EGFR)-sensitive mutations can be treated with small-molecule tyrosine kinase inhibitors (TKIs) as first-line therapy. However, drug resistance inevitably develops within 10-13 months (3, 4). The resistance mechanisms of EGFR TKIs mainly include T790M mutation, cell-mesenchymal epithelial transition factor (C-MET) and human epidermal growth factor receptor 2 (HER2) gene amplification, kirsten rat sarcoma viral oncogene (KRAS) gene mutation, vrafmurine sarcoma viral oncegene homolog B (BRAF) gene mutation, and SCLC transformation (5–9). Some drug-resistant cases can be overcome by replacing the corresponding targeted drugs with better results, but the transformation of the pathological type brings great confusion to the clinical diagnosis and treatment process. In this paper, we report a case of advanced lung adenocarcinoma with EGFR-sensitive mutation that was transformed into small-cell lung cancer after EGFR TKIs treatment. The histological transformation from adenocarcinoma to small cell lung cancer may involve phenotypic changes in tumor cells, which could be related to genetic variations and clonal selection within the tumor. Given the heterogeneity and dynamic nature of the tumor, continuous monitoring and personalized treatment are crucial for these patients. Therefore, in the subsequent treatment process, we repeatedly performed needle biopsies and guided treatment decisions based on the pathological results. The patient received anlotinib-containing therapy together with chemotherapy and/or immunotherapy after transformation to achieve survival benefit. The case is reported as follows, along with a literature review for clinical reference.

## Case presentation

A 48-year-old male patient with no history of smoking or alcohol consumption. He was diagnosed with right lung adenocarcinoma cT2N0M1a (pleural metastasis) stage IV A in June 2020, with EGFR 19 exon deletion mutation, negative for anaplastic lymphoma kinase (ALK), KRAS, proto-oncogene 1, receptor tyrosine kinase (ROS1), MET, BRAF. Gefitinib was started on June 3, 2020 and continued with good response.

**Figure 1:**
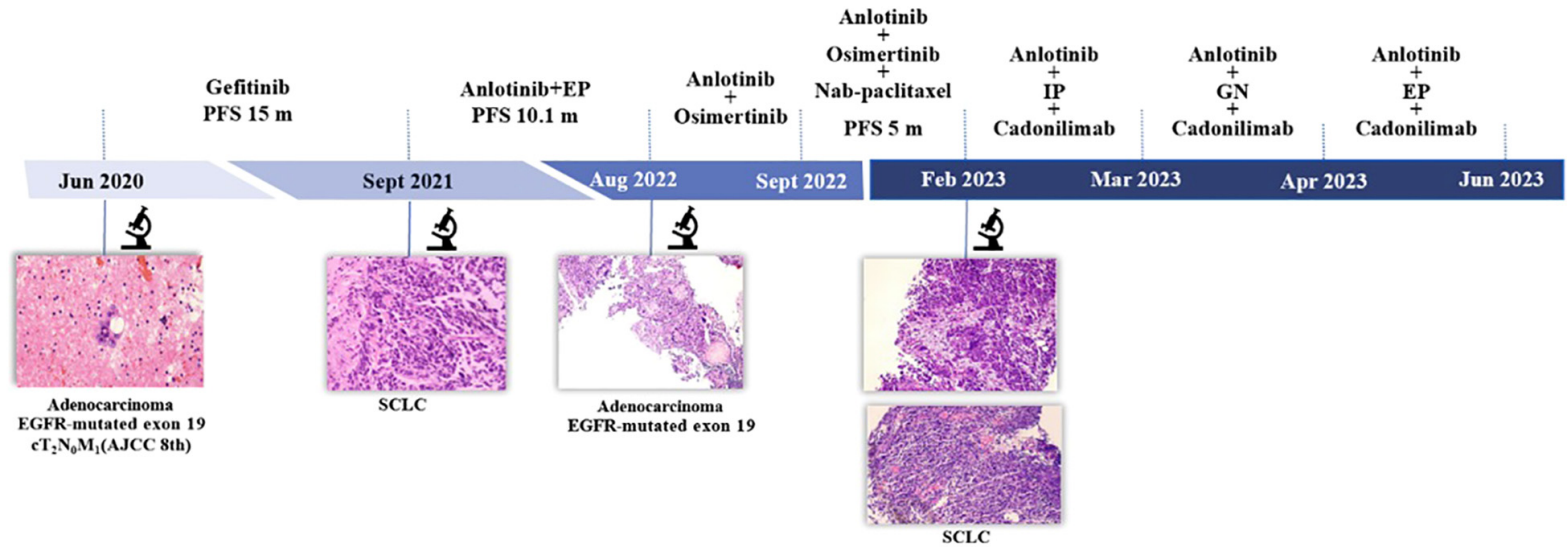
Timeline. AJCC 8^th^, American Joint Committee on Cancer; EP, etoposide and cisplatin; IP, irinotecan and cisplatin; GN, gemcitabine and vinorelbine; PFS, progression-free survival; SCLC, small cell lung cancer.

**Figure 2:**
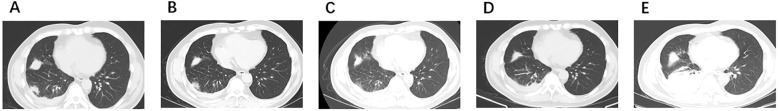
Radiologic images. **(A)** September 2021: SCLC transformation. **(B)** November 2021: PR to anlotinib combined with EP. **(C)** February 2022: PR to anlotinib combined with EP. **(D)** June 2022: SD to anlotinib. **(E)** August 2022: PD to anlotinib. PR, partial response; SD, stable disease; PD, progressive disease.

**Figure 3:**
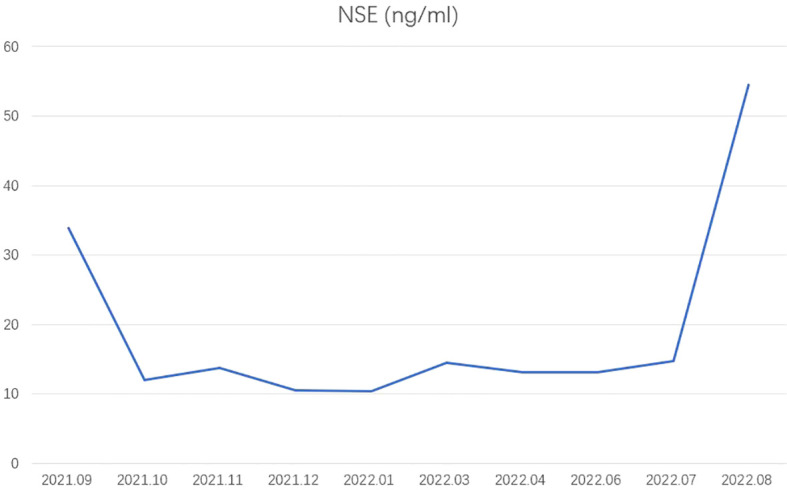
Tumor markers. NSE, neuron-specific enolase.

Disease progression was confirmed in September 2021. Subsequently, right lung biopsy was performed and pathological diagnosis was small cell carcinoma with immunohistochemistry: Pan-cytokeratin (PCK) (partial +), chromogranin A (CgA) (+), synaptophysin (Syn) (+), neural cell adhesion molecule-1 (NCAM-1/CD56) (+), thyriod transcription factor-1 (TTF-1) (+), cytokeratin-7 (CK7) (-), napsinA (-), Ki-67 (Li:70%). Etoposide and platinum (EP) regimen is commonly used in the treatment of classical SCLC. Meanwhile, considering the poor treatment efficacy and rapid disease progression often observed after SCLC transformation, we choose the treatment regimen of EP combined with anlotinib, an oral tyrosine kinase inhibitor. The patient started treatment with anlotinib together with EP regimen on October 2, 2021. After six cycles of anlotinib in combination with EP, patients with a partial response received oral anlotinib as maintenance therapy (**Figures 1**–**3**).

In August 2022, due to disease progression (axillary lymph node metastasis), a right axillary lymph node biopsy was performed, which revealed metastatic adenocarcinoma with EGFR19 deletion mutation along with phosphatidylinositol-4, 5-bisphosphate 3-kinase, catalytic subunit alpha (PIK3CA) mutation, tumor protein p53 (TP53) mutation, phosphatase and tensin homologue deleted on chromosome 10 (PTEN) deletion, retinoblastoma1 (RB1) deletion. According to the mutation of EGFR 19 deletion, anlotinib together with osimertinib were given on August 11, 2022. After a short period of 21 days, the significant reduction in axillary lymph nodes, but a new metastasis was found. However, the new lesion was too small to be biopsied. Chemotherapy, nab-paclitaxel, was added to the previous regimen of anlotinib and osimertinib for this patient who had a good performance status. This combination was proven to have a good response and had been given for 6 cycles.

In February 2023, two new lesions were confirmed as new metastases with small cell carcinoma both in the right lower lung and left supraclavicular lymph node. We tried several chemotherapy regimens along with anlotinib and cadonilimab, but all of these regimens failed to achieve a response in this patient, and he died on June 9, 2023 (**Figure 1**).

## Discussion

SCLC transformation is one of the mechanisms of drug resistance in TKIs-treated NSCLC patients with EGFR-sensitive mutations, and relevant studies have shown that the incidence of SCLC transformation is 5% ~ 14%, which generally occurs 14-26 months after TKIs treatment, and the median time of transformation is 18 months (4, 10–12). Transformed SCLC shares similarities with classical SCLC in terms of pathology, clinical manifestations, and drug sensitivity, but cannot be fully classified as classical SCLC (13). Transformed SCLC is often poorly treated with rapid disease progression, and the standard chemotherapy regimen for SCLC, EP, is the mainstay of treatment. Marcoux et al. retrospectively found (14) that treatment with etoposide in combination with a platinum regimen after SCLC transformation resulted in a clinical response rate of 54%, with a median progression-free survival (mPFS) of 3.4 months. Median overall survival (mOS) after SCLC transformation was only 10.9 months as reported. Transformed SCLC after resistance to third-generation EGFR TKIs had a better prognosis than resistance to first- and second-generation EGFR TKIs (12). In this case, the treatment regimen of anlotinib combined with EP in the transformation of SCLC after resistance to first-generation EGFR TKIs therapy made the patient have a good clinical outcome with PFS of 10.1 months, and the OS more than 36 months.

The mechanism of SCLC transformation is uncertain. Current mechanisms involved in SCLC transformation include lineage plasticity and tumor heterogeneity. (I) Lineage plasticity is the ability of cells to transdifferentiate from one fixed lineage to another. Inhibition of EGFR signaling by TKIs can selectively promote histologic transformation in tumor cells with specific genomic backgrounds (e.g., TP53 mutations and RB1 deletions), i.e., from epithelial lineage adenocarcinoma to neuroendocrine lineage SCLC. (II) Pre-treatment tumor heterogeneity. Nicholson et al. (15) analyzed a cohort of 100 SCLC patients and found that 28% of SCLC cases were of mixed histology. Zhao et al. (16) also found 10 cases of SCLC combined with mixed histology in a cohort of 170 SCLC patients. These results support the theory that pre-treatment tumor heterogeneity leads to transformation to SCLC. In our case, the patient’s initial diagnosis was established through pleural fluid cytology without needle biopsy of the primary site. Upon demonstrating resistance to EGFR-TKI therapy, a needle biopsy of the primary site subsequently identified the presence of small cell lung cancer. As treatment progressed, puncture biopsies conducted at various metastatic sites revealed the coexistence of both adenocarcinoma and small cell carcinoma. The possible factors leading to this phenomenon include: (I) Tumor Heterogeneity: Within the tumor, there may exist different subpopulations of cells that vary in genetics, epigenetics, or phenotype. This heterogeneity can lead to different metastatic sites exhibiting distinct pathological features. (II) Lineage Plasticity: Cancer cells may transition from adenocarcinoma to small cell carcinoma, possibly to adapt to a new microenvironment or evade treatment. (III) Genetic and Epigenetic Variations: Tumor cells may accumulate genetic and epigenetic variations during growth and metastasis, which can lead to changes in cell phenotype. For example, mutations in certain genes or alterations in epigenetic modifications can cause adenocarcinoma cells to transform into small cell carcinoma cells. (IV) Influence of the Microenvironment: The microenvironment in which tumor cells reside can significantly impact their phenotype. Different microenvironments may promote or inhibit the growth and differentiation of specific types of cancer cells, resulting in metastatic sites exhibiting different pathological types. (V) Treatment-induced Adaptive Changes: Treatment can exert selective pressure on tumor cells, leading to the emergence of drug-resistant subpopulations. These resistant cells may have different phenotypes, including a transition from adenocarcinoma to small cell carcinoma. It was found that RB1 deletion and TP53 mutation were prevalent in patients who developed SCLC transformation, and the risk of SCLC transformation in patients with EGFR-mutated lung adenocarcinoma combined with RB1 deletion and TP53 mutation was 42.8 times higher than that in patients without RB1 deletion and TP53 mutation (13). In clinical practice, NSCLC patients with RB1 and TP53 inactivation need to be aware of the possibility of SCLC transformation during treatment. In addition, myelocytomatosis viral oncogene homolog (MYC) amplification, Notch pathway activation, Achaete-scute homolog-1 (AscL1) gene expression, PIK3CA mutations, and catalytic polypeptide hypermutation by apolipoprotein B (ApoB) mRNA editing enzyme have been implicated in SCLC transformation (11, 17–20).

Anlotinib is an oral tyrosine kinase inhibitor that can effectively inhibit the kinases of vascular endothelial growth factor receptor (VEGFR), platelet-derived growth factor receptor (PDGFR), fibroblast growth factor receptor (FGFR), stem cell factor receptor (c-Kit), etc. Anlotinib has the potential to be an anti-kinase and has the ability to inhibit the kinases of FGFR and c-Kit, which have dual roles of anti-tumor angiogenesis and tumor growth inhibition (21–23). Anlotinib has a potent inhibitory effect on VEGFR, PDGFR and FGFR signaling pathways. Therefore, compared with other anti-angiogenic drugs, anlotinib can simultaneously inhibit the three signaling pathways related to angiogenesis and comprehensively block tumor neovascularization. c-Kit-mediated signaling pathway plays an important role in the initiation, development and recurrence of many malignant tumors, and anlotinib has also shown strong inhibitory activity on c-Kit. Currently, some studies indicated that anlotinib combined with etoposide and platinum as first-line treatment for extensive-stage SCLC also showed good efficacy and tolerability (24, 25). Hu et al. (24) showed that the mPFS of anlotinib combined with platinum-etoposide chemotherapy in the first-line treatment of extensive-stage SCLC was up to 8.02 months, and the mOS was up to 15.87 months. A Chinese multicenter real-world study (25) showed that the mPFS was 6.0 months and the mOS was 10.5 months. In this case, we innovatively adopted the treatment regimen of anlotinib combined with EP in the transformation of SCLC after resistance to EGFR TKIs therapy, and the PFS reached 10.1 months, which was a longer PFS compared with the data from previous case reports and retrospective analyses, suggesting that the anlotinib combined with EP regimen could be the first choice of treatment after transformation to SCLC. This case was transformed to SCLC after treatment of a first-generation EGFR TKIs, and the combination treatment modality (chemotherapy, target therapy and immunotherapy) with anlotinib was continued in the subsequent treatment, which enabled this patient to have an OS of more than 20 months after transformation to SCLC.

A study by Wang et al. (26) retrospectively analyzed 29 patients with transformed SCLC. Except for one patient who died shortly after transformation, the other 28 patients received at least one treatment after transformation. Sixteen patients received chemotherapy in combination with EGFR TKIs and 8 patients received chemotherapy without EGFR TKIs. Compared to chemotherapy without EGFR TKIs, chemotherapy with EGFR TKIs improved mPFS (5.2 months vs. 3.0 months, p=0.014). However, there was no significant effect on mOS (14.8 vs. 13.0 months, p=0.474). Thirteen patients received local radiotherapy and the results showed that radiotherapy improved OS in patients who transformed to SCLC compared to no radiotherapy (15.5 months vs. 13.9 months, p=0.045). In addition, 18 patients received anti-angiogenesis inhibitor monotherapy or combination therapy, of which 83.3% were treated with anlotinib, 6 patients were treated with anlotinib monotherapy, and the rest were treated with chemotherapy and/or EGFR TKIs. The results showed that anti-angiogenesis inhibitors significantly improved OS in patients who converted to SCLC. In another retrospective study by Wang et al. (12), 5 patients with transformed SCLC were treated with anlotinib, with an objective response rate (ORR) of 66.7% and a mPFS of 6.2 months, which also showed the efficacy of anlotinib in the treatment of transformed SCLC (**Table 1**).

**Table 1:** Literature review of small cell lung cancer transformation.

Author	Publication type	Sample size	Treatment	Clinical Outcomes				
				ORR (%)	DCR (%)	mPFS (months)	mOS (months)	
Wang (12)	Review	27	EP	44.4	74. 1	3.5	9.7 (8.4- 10.9)	
		3	IP	66.7	100.0	7.6		
		5	Anlotinib	66.7	80.0	6.2		
Marcoux (14)	Review	53	EP	54.0	N/A	3.4	10.9 (8.0- 13.7)	
		21	TAX	50.0	N/A	2.7		
Wang (26)	Review	16	Chemotherapy with EGFR-TKIs	43.8	N/A	5.2	14.8	P=0.474
		8	Chemotherapy without EGFR-TKIs	37.5	N/A	3.0	13.0	
		18	Anti-angiogenesis	N/A	N/A	N/A	15.1	P<0.001
		10	Non-anti-angiogenesis	N/A	N/A	N/A	4.3	
		13	Local radiotherapy	N/A	N/A	N/A	15.5	P=0.045
		15	Non-radiotherapy	N/A	N/A	N/A	13.9	
LAI (31)	Case reports	1	Erlotinib and EP	N/A	N/A	3.0	N/A	
		1	Erlotinib and EP followed by oral etoposide	N/A	N/A	8.0	N/A	

DCR, disease control rate; EP, etoposide and platinum; IP, irinotecan and platinum; mPFS, mediam progression free survical; mOS, median overall survival; N/A, not available; ORR, objective response rate; TAX, paclitaxel.

The efficacy of immunotherapy in patients with SCLC transformation has been unclear. Sehgal et al. (27) showed that transformed SCLC patients treated with nivolumab or pembrolizumab monotherapy could achieve a mOS of 13.0 months after transformation to SCLC, suggesting that immunotherapy may provide a survival benefit to transformed SCLC patients. However, a retrospective study showed that immunotherapy were ineffective in 17 patients with SCLC transformation (14). Related studies have found that EGFR-mutant NSCLC often presents an immunosuppressive phenotype, while most SCLC are also immunosuppressive with a lack of major histocompatibility complex-I (MHC-I) expression, and transformed SCLC also exhibit reduced MHC-I expression (28–30). The present patient also received immunotherapy, but it did not appear to be beneficial. Therefore, immunotherapy for transformed SCLC needs to be further investigated at this time.

## Conclusion

Based on the pathological findings, the case presents a complex scenario of tumor heterogeneity and transformation. This case highlights the importance of continuous monitoring and re-evaluation of tumor characteristics, as well as the challenges in managing patients with evolving tumor biology. The differential diagnosis and tailored treatment strategies are crucial in such cases to address the diverse therapeutic needs arising from the presence of different histological subtypes within the same patient.

This case of advanced lung adenocarcinoma patient was transformed to SCLC resistant to one generation EGFR TKIs treatment, and the front-line treatment after transformation was a regimen of anlotinib in combination with EP, which resulted in the patient achieving a long PFS. After the first progression with anlotinib in combination with EP, anlotinib was shown to be effective with nab-paclitaxel. Subsequent treatment with an anlotinib-containing regimen resulted in an OS of more than 20 months for the patient. It is suggested that for transformed SCLC patients, the treatment containing anlotinib may provide survival benefit and is a feasible option.

## Data Availability

The original contributions presented in the study are included in the article/supplementary material. Further inquiries can be directed to the corresponding authors.
